# Interactions and Diffusion of Methane and Hydrogen in Microporous Structures: Nuclear Magnetic Resonance (NMR) Studies

**DOI:** 10.3390/ma6062464

**Published:** 2013-06-17

**Authors:** Yu Ji, Neil S. Sullivan, Yibing Tang, Jaha A. Hamida

**Affiliations:** Department of Physics, University of Florida, Gainesville, FL 32611, USA; E-Mails: charyu@hotmail.com (Y.J.); tangyb@phys.ufl.edu (Y.T.); jaha1953@yahoo.com (J.A.H.)

**Keywords:** nuclear resonance, spin-lattice relaxation, heat capacity, mesoporous structures

## Abstract

Measurements of nuclear spin relaxation times over a wide temperature range have been used to determine the interaction energies and molecular dynamics of light molecular gases trapped in the cages of microporous structures. The experiments are designed so that, in the cases explored, the local excitations and the corresponding heat capacities determine the observed nuclear spin-lattice relaxation times. The results indicate well-defined excitation energies for low densities of methane and hydrogen deuteride in zeolite structures. The values obtained for methane are consistent with Monte Carlo calculations of A.V. Kumar *et al.* The results also confirm the high mobility and diffusivity of hydrogen deuteride in zeolite structures at low temperatures as observed by neutron scattering.

## 1. Introduction

**Figure 1:**
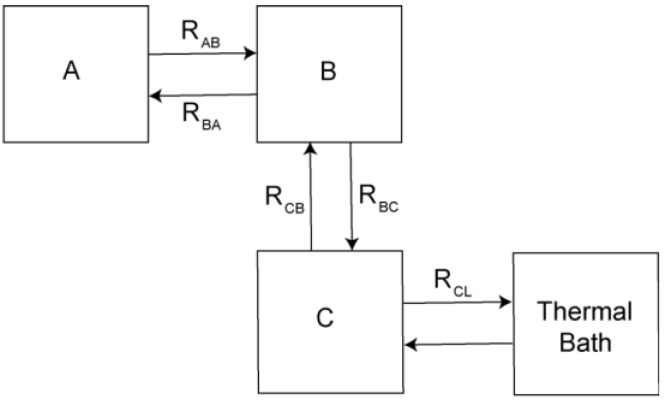
Schematic representation of three energy baths A, B, and C with a weak link (bottleneck) between B and C with energy flow from C to the thermal bath. In this example, there is a bottleneck in the vertical path R_BC_.

There is currently wide interest in the thermodynamic properties of light gases constrained to the interior of microporous structures that include metal organic frameworks [1,2,3] and classical zeolitic structures [4] because of the potential use of these structures for storage and transport [5,6,7,8], and catalytic conversion [9] of light molecular gases (H_2_, CH_4_, CO_2_, CO, *etc.*). In addition to these practical applications, studies of these systems are of special fundamental interest for exploring the novel properties of quantum fluids when the constraining geometry has dimensions comparable to the thermal de Broglie wavelength or the thermal phonon wavelength [10,11]. While there is a large body of information about the total adsorption of light gases in many microporous structures, much less information is available about the strength of the interactions of the molecules with the confining walls, and detailed dynamics [12] of the gases inside the porous materials. Of particular interest is the thermal activation of diffusion from one micropore to a neighboring pore. We describe a novel application of some well known nuclear spin-lattice relaxation processes that are relevant when the excitation of the molecular systems can be described in terms of coupled thermal reservoirs associated with the internal degrees of freedom of the molecules (molecular rotations, quantum tunneling, *etc.*) that have well defined but weak inter-connections and weak nuclear spin couplings to the lattice or between different degrees of freedom. This multiple bath model has been used to describe the nuclear spin-lattice and spin-spin relaxation times in solid ^3^He at low temperatures [13], solid deuterium at intermediate temperatures [14], and the diffusion of hydrogen deuteride impurities in solid hydrogen [15,16]. As a general example we will follow the arguments of Guyer, Richardson, and Zane [13], and consider three energy baths A, B, and C whose internal degrees of freedom can be considered as quasi-independent (e.g., molecular rotations) and that are weakly coupled to one another while only one of the energy baths is strongly coupled to the lattice, as illustrated in Figure 1.

If these energy baths (A, B, C) can reach internal thermal equilibrium on a time scale smaller than that of the couplings between the baths, the observed overall relaxation will depend on the heat capacities of the individual baths. In particular, if as in Figure 1, the rate R_BC_ is the weakest (slowest) thermal link, the observed relaxation rate is given by

$$T_{1,observed}=\frac{C_{A}+C_{B}}{C_{B}}T_{BC}$$

where $T_{BC}=R_{BC}^{-1}$ . A detailed derivation of this expression and the corresponding amplitudes of the components of the relaxation is given in Appendix A. We first review the application of this bottlenecked relaxation for the cases of solid ^3^He, and for light molecular gases (hydrogen deuteride and CH_4_) trapped in zeolitic structures.

In the case of solid ^3^He (which is well understood [13]), A is the nuclear spin system, B represents the excitations due to atomic motion (quantum tunneling motions and/or vacancy motion), and C is the phonon system for solid ^3^He, which is in contact with the containing wall, which in turn is linked to the thermal bath. Following a perturbation of the nuclear spin system (bath A) with, for example, the application of an RF pulse, the thermal bath A will come to a common internal temperature (a nuclear spin temperature) in a time ${\left(M_{2}\right)}^{-\frac{1}{2}}≃0.1$ ms where *M*_2_ designates the second moment of the internal nuclear dipole-dipole interactions [13]. The transfer of energy from bath A to bath B (the tunneling degrees of freedom) is in typical cases much slower (~0.01–0.1 s) because the atom-atom exchange frequencies are much higher than the Zeeman frequency for modest magnetic fields. The weakest link at low temperatures (T ~ 0.1–0.3 K) is the connection between the tunneling motions and the phonons of the ^3^He lattice because of the enormous difference between the tunneling energies (~mK) and the phonon energies (10–30 K). Typical time constants for this energy exchange are ~1.0–100 s [13]. In this scenario, the observed relaxation is a function of both the intrinsic relaxation rates and the internal heat capacities of the different baths.

For solid ^3^He,

$$C_{A}=\frac{1}{2}Nk_{B}{\left(ћ\omega_{L}/k_{B}T\right)}^{2}$$

where *k_B_* is Boltzmann’s constant, *T* is the absolute temperature, and $\omega_{L}$ is the nuclear Larmor frequency.

$$C_{B}=\frac{3}{8}zNk_{B}{\left(ћJ/k_{B}T\right)}^{2}$$

where *J* is the exchange frequency due to quantum tunneling and $C_{C}=234Nk_{B}{\left(T/{\text{Θ}}_{D}\right)}^{3}$ where ${\text{Θ}}_{D}$ is the Debye temperature for solid ^3^He. (We use the low temperature limit for the phonon heat capacity.)

**Figure 2:**
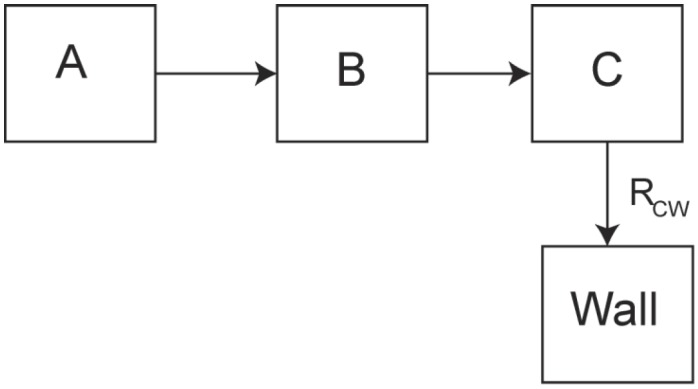
Multiple bath model for relaxation of methane molecules in zeolite cages. A represents the nuclear spin energies plus the ground rotational state, B represents the excited rotational states (only the T states have nuclear spin), C consists of the translational molecular motions, and the wall represents the excitations (phonons) of the zeolite lattice. If R_CW_ is the thermal bottleneck, the observed relaxation time is $\tau_{Obs}=(1+\frac{C_{A}+C_{B}}{C_{C}})R_{CW}^{-1}$ .

For methane, in addition to the nuclear spin degrees of freedom (bath A) we need to consider the rotational motion (bath B) and translational motion (bath C) of the molecules and their thermal contact with the wall (D) of the zeolite (phonons of zeolite), shown in Figure 2. For bath A, as in the case of solid ^3^He, the nuclear spins reach internal equilibrium on a time scale of ~0.1 ms as determined by the nuclear spin-spin interactions. The coupling between baths A and B is determined by the spin-rotation coupling $H_{SR}=CIJ$ with *C* = 42 kHz [17]. *I* is the nuclear spin, and *J* the rotational angular momentum. The AB coupling time constant is found to be ~0.1 ms, and baths A and B come to a common temperature very rapidly. The same is also true for the coupling between baths B and C, which is determined by electric octupole-octupole interaction between molecules. This octupolar coupling is given by *H_OO_* = *I*^2^/*R*^7^ [17] where I is the electric octupole moment and R the separation of the molecules. For solid methane *R* = 4.6 Å and *H_OO_* ~ 3 K. For well-separated molecules in zeolite-like structures, R ~ 10 Å and *H_OO_* ~ 10^−3^ K. On expanding *H_OO_* in terms of small displacements we find a coupling time constant *τ_BC_* ~ 10 ms.

The large bottleneck at low temperatures is between the translational modes of the molecules and the phonons in the wall of the zeolite. This Kapitza resistance at the wall of the mesoscopic structures results from the large difference between the velocity of translational modes of methane (*ν_t_* ~ 200 m/s) compared to those of the solid zeolite, *ν_s_* ~ 5000 m/s. If *ρ_t_* and *ρ_s_* are the respective densities of the methane and zeolite atoms, only a fraction $f\sim \frac{\rho_{t}}{\rho_{s}}{\left(\frac{v_{t}}{v_{s}}\right)}^{3}\sim 10^{-5}$ of the translational excitations of the CH_4_ can transfer energy to the phonons in the wall, leading to thermal time constants of 0.1–1.0 s.

For simplicity in the case of methane we include in bath A both the nuclear Zeeman energy and the ground state for the rotational degrees of freedom. There are three molecular states for a methane molecule, *A*, *T*, and *E*, corresponding to the different spin-symmetry configurations for a tetrahedral group of fermions. The *E* state has no total nuclear spin and plays no role in the nuclear relaxation processes. The excited states are therefore given by the *T* species, of which there are at least two, *T*_1_ and *T*_2_, corresponding to the two different symmetry sites in a zeolite cage. The nuclear Zeeman energy and the rotational ground state of the *A* species are considered as one single bath because they are tightly coupled. The heat capacity for bath B is the sum of the contributions for the *T*_1_ and *T*_2_ excitations, with $C=\sum_{i=1,2}g_{i}N_{i}k_{B}{\left({\text{Δ}}_{i}/T\right)}^{2}\exp(-{\text{Δ}}_{i}/T)/{\left(1+\exp(-{\text{Δ}}_{i}/T)\right)}^{2}$ where ∆*_i_* are the excitation energies for the *T*_1_ and *T*_2_ states and *g_i_* are the relative degeneracies.

For hydrogen deuteride molecules trapped in mesoporous structures, A is the combined bath for the Zeeman and the ground state for the translational degrees of freedom, and B is the bath corresponding to the excited states for the hydrogen deuteride molecules (wavenumber non-zero). In this case, C_A_ is expected to be the low temperature Debye heat capacity (for the ground state) and C_B_ is the sum of the Schottky heat capacities for each excited energy level.

## 2. Experimental Section

**Figure 3:**
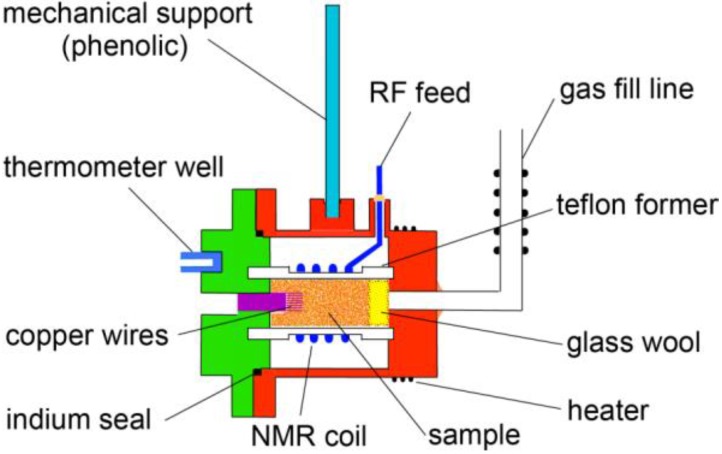
(Color on line) Schematic illustration of sample cell showing NMR coil and thermal linkage to sample from cold cap (green) and copper shroud (red).

**Figure 4:**
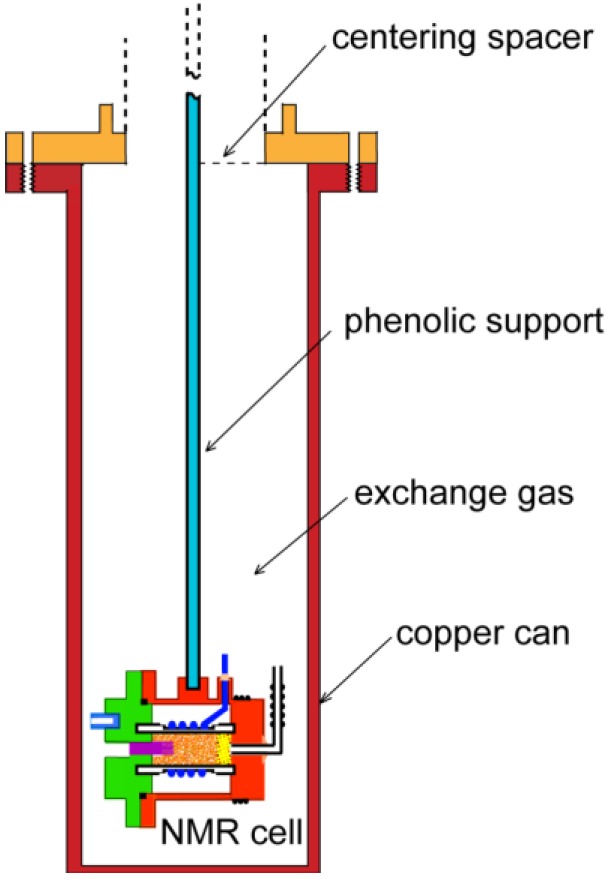
(Color on line) Schematic representation of the support structure and cooling path for the sample cell. The exterior copper can sits in a liquid helium bath that can be pumped to 1.4 K.

**Figure 5:**
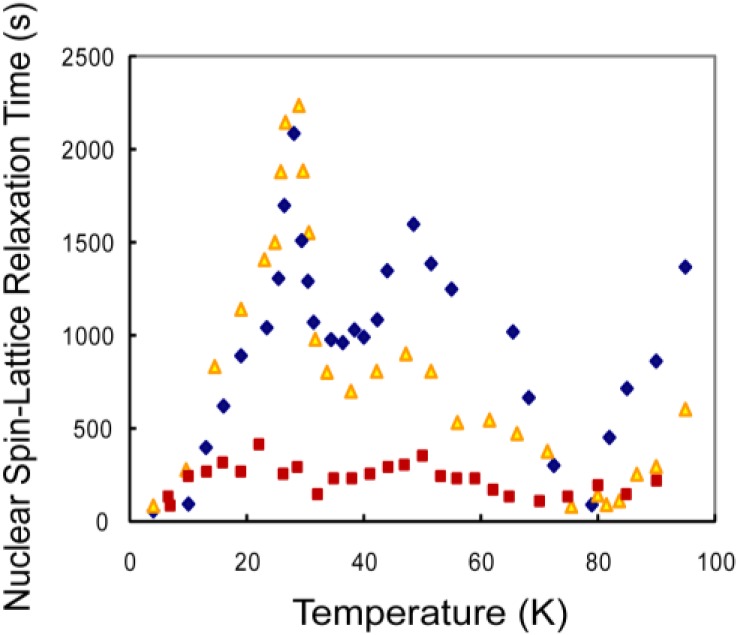
(Color on line) Variation of the CH_4_ proton spin-lattice relaxation with temperature for molecules confined to α-cages of zeolite: diamonds, 1.0 molecule per cage; triangles, 0.5 molecules per cage; and squares, 0.05 molecules per cage. The Shottky dependence is lost for low filling factors.

The samples of zeolite-13X (Na_86_[(AlO_2_)_86_(SiO_2_)_106_]·264H_2_O) were prepared from commercially available material [18] that was in the form of hard pellets (1.5 mm outer diameter). The pellets were lightly crushed to facilitate gas penetration and then activated by heating to 200 °C in a high vacuum (<10^−5^ Torr.) for seven hours. Following activation, nitrogen adsorption isotherms were measured [19] to characterize the sample and to check that the expected surface area per unit mass was realized. The samples were then placed inside a Teflon^®^ [20] coil former that was inserted into a groove in a cold finger, Figure 3. The end of the Teflon^®^ chamber was sealed with a plug of glass wool to prevent motion of the crushed zeolite while providing for gas entry. The sample cell was enclosed by a copper vacuum shroud using an indium metal seal to ensure vacuum integrity at low temperatures. A short brush of very fine copper wires (0.1 mm outer diameter) provided the thermal linkage from the sample to the copper enclosure. The latter had a calibrated carbon glass thermometer affixed to the copper cold block (Figure 4) to measure the temperature. A twisted bifilar heater was wound around the cell to regulate the temperature using a current derived from an error signal between the thermometer readout and a desired temperature determined by a setting on a resistance bridge. Helium exchange gas was admitted to the space separating the metal shroud from a liquid helium bath (Figure 5) to provide a weak thermal link from the helium bath to the sample cell. The cell itself was suspended by an insulating rod fabricated from bakelite. This design allowed us to hold the temperature to within 0.1% of the set temperature for 2 < T < 150 K.

In order to determine the precise coverage for methane on the interior surfaces of the zeolite we measured the adsorption isotherm for methane (on the sample) at T = 77 K. The results for the isotherm studies have been reported elsewhere [21] and are summarized in Appendix B. The isotherm exhibits a distinct jump at the value of adsorbed gas corresponding to the saturation of the α-cages of zeolite-13X for 1.1 × 10^−2^ mol/g. The latter was estimated from the mass of the sample and the known surface area of zeolite-13X of 660–800 m^2^/g [18,22].

A pulse NMR spectrometer was used to measure the nuclear spin relaxation times. A relatively low frequency (5.5 MHz, corresponding to the proton nuclear Larmor frequency in an applied magnetic field of 0.129 T) was chosen to realize the condition for which the Larmor frequency matched the anticipated tunneling rate for one molecule to pass from one cage to another as estimated by recent Monte Carlo calculations [12]. This condition allows one to observe the Bloembergen-Purcell-Pound [23] minimum in the relaxation time from which one can infer the tunneling rate directly in addition to the thermal activation energy. The NMR coil was connected via a low-loss cryogenic cable to a fast-recovery RF duplexer similar to the design of Deschamps *et al.* [24,25], but altered to work with a high impedance parallel resonance circuit. Two different pulse sequences were employed: (i) 90*_x_*–90*_y_* and (ii) 90*_x_*–180*_x_* sequences in order to observe solid and liquid-like echoes, respectively, with the latter appropriate to the “quasi-melting” behavior expected at the temperatures corresponding to thermal activation of intercage diffusion. Because of the low filling factors employed, typically less than one molecule per cage, the signal to noise ratios are weak and the signals were therefore accumulated and averaged using a computer interfaced digital storage oscilloscope.

## 3. Results and Discussion

### 3.1. CH_4_ Molecules

The values of the relaxation times measured for different quantities of CH_4_ adsorbed on zeolite-13X are shown in Figure 5. Three features are observed: two peaks at 27 K and 46 K, and a deep minimum at 78 K. The two peaks are attributed to two tunneling states for the *T* molecular species associated with two different sites for localization of the CH_4_ molecules. In the two-bath model, bath B consists of the thermal excitations to the distinct rotational states *T*_1_ and *T*_2_. The ground state for the molecular rotations referred to in the literature as the symmetric *A* state with the nuclear spin excitations forms bath A. The heat capacity for bath B therefore consists of two Shottky-like heat capacities giving rise to the two peaks in the observed relaxation times. The values of the energy peaks (27 K and 49 K) are close to those reported for earlier heat capacity measurements that were seen as weak bumps in the total heat capacity [26]. The peaks in the observed relaxation times are much sharper than those for simple Shottky heat capacities and this is expected because the excited states are expected to be pocket states that undergo small amplitude librational motion about their equilibrium orientations.

**Figure 6:**
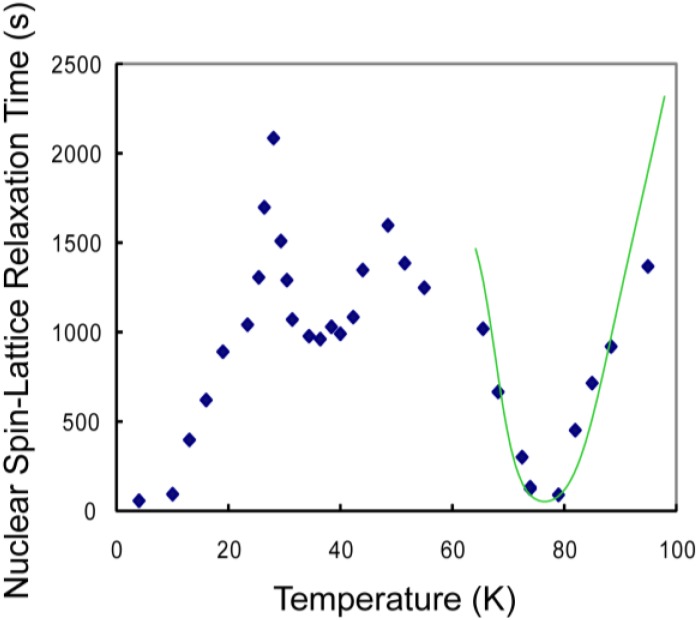
(Color on line) Fit of variation of the observed relaxation time with temperature for CH_4_ molecules in zeolite-13X at 78 K to thermal activation of intercage diffusion for 0.5 molecules per cage. The solid green line corresponds to an activation energy of 20.8 ± 1.5 kJ/mol. and a microscopic tunneling rate of 1.2 × 10^15^ s^−1^.

The broad minimum at high temperatures is interpreted as a classic Bloembergen-Purcell-Pound minimum [23] associated with a thermally activated diffusion for passage from one α-cage to adjacent cages with an intercage tunneling of $\tau =\tau_{0}\text{exp}(-\frac{E_{A}}{k_{B}T})$ . The fit shown in Figure 6 yields an activation energy *E_A_* = 20.8 ± 1.5 kJ/mol. and a tunneling rate τ_0_ = 1.2 × 10^15^ s^−1^. This value deduced for the activation energy is comparable to the value of 22 kJ/mol. estimated from Monte Carlo studies [27].

### 3.2. Hydrogen Deuteride Molecules

In order to carry out a more fundamental study of the molecular relaxation and diffusion in mesoporous structures we carried out experiments on hydrogen deuteride trapped in cages of zeolite. While methane has several distinct molecular species (ortho, meta, and para), corresponding to the different combinations of rotational symmetry and nuclear spin symmetry, hydrogen deuteride molecules do not have these properties and can be regarded as spherical molecules with a weak electric dipole moment. We chose hydrogen deuteride rather than H_2_, because like CH_4_, H_2_ has two molecular species, ortho-H_2_ (with total nuclear spin I = 1 and orbital angular momentum J = 1) and para-H_2_ (with I = 0 and J = 0). Only ortho-H_2_ can be observed by nuclear magnetic resonance and this species converts to para-H_2_ slowly via magnetic interactions. In addition, ortho-H_2_ has an electric quadrupole moment that results in interesting molecular alignment configurations, but these can be difficult to interpret [28]. The relaxation of hydrogen deuteride molecules, however, is determined by their translational degrees of freedom and the spin-spin interactions between these molecules and the interactions with the walls of the cages. Hydrogen deuteride experiments are therefore ideal for determining the molecular diffusion in mesoporous materials and for exploring the effects of confinement on the molecules. The experiments can test if the translational degrees of freedom are quantized as expected for a perfect spherical cage.

**Figure 7:**
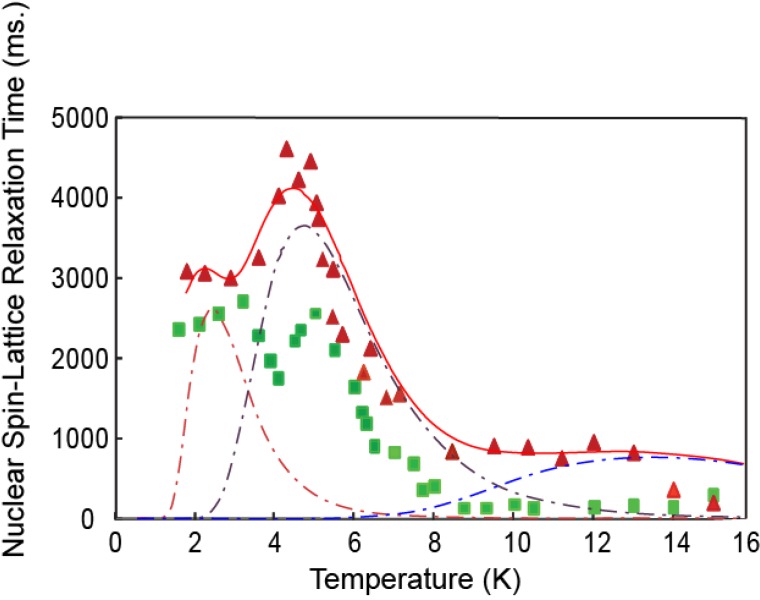
Observed variation of the hydrogen deuteride proton spin-lattice relaxation with temperature for molecules adsorbed in zeolite 13× with coverages of 1.0 molecule per cage (squares) and 0.5 molecules per cage (triangles). The solid line shows the contribution assuming Shottky level specific heats for three discrete energy levels. Each contribution is shown by the broken lines.

**Figure 8:**
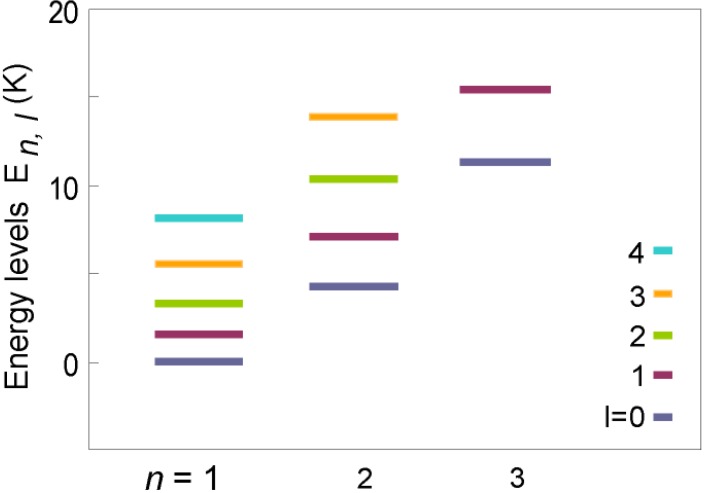
(Color on line) Schematic representation of the translational energy levels *E_l,n_* for hydrogen deuteride molecules constrained to a cage 15 Å in diameter.

Figure 7 shows the values of the CH_4_ proton spin-lattice relaxation times observed for temperatures 1.5 < T < 15 K for two different densities, (i) 1.0 molecule per cage and (ii) 0.5 molecules per cage. The densities were determined from the adsorption isotherms [21]. Distinct peaks are seen for each filling, *x*, but at different temperatures for the different fillings: at 2.9 K, 4.9 K and 7.3 K for *x* = 1.0; and at 2.1 K, 4.5 K and 12.3 K for *x* = 0.5. In Figure 8 we show the fit assuming distinct Shottky heat capacity contributions (illustrated by the broken lines) for the data for 0.5 molecules per cage. The Shottky heat capacity form $C_{i}=Nk_{B}{\left(\frac{{\text{Δ}}_{i}}{T}\right)}^{2}\exp(-\frac{{\text{Δ}}_{i}}{T})/{\left[1+\exp(-\frac{{\text{Δ}}_{i}}{T})\right]}^{2}$ (for each excited state ∆*_i_*) can only be considered as an approximation for the weakly trapped molecules and is valid only if the excited states can, to a good approximation be regarded as discrete energy levels. In the bath model of Figure 2, $C_{B}=\sum_{i}C_{i}$ for the *i* excited states, and *C_C_* is the low temperature heat capacity for the molecular translational motions. In Figure 7 we have assumed an arbitrary amplitude for each contribution to fit the data. The values for the excitation levels shown in Figure 7 are very different from the principal adsorption energies of ~80 K and ~40 K, respectively, for the S1 and S2 bonding sites for hydrogen in zeolite-13X as determined from neutron scattering experiments [29,30]. The S1 sites are at the centers of the six-membered rings adjacent to the eight-sided opening of the α-supercages of the zeolite structure, and the S2 sites are located close to the octagonal openings of the α-supercages. For a detailed summary of the positions of these sites relative to the supercages the reader is referred to Figure 6 of [29], Figure 2 of [31], and Figure 2 of [32]. These discrete energies could be due to: (i) small energy barriers between the potentials at the S1 and S2 sites that allow molecules to jump form one site to another; and (ii) a number of closely spaced energy levels inside the binding potentials.

### 3.3. Discussion

If we consider a simple molecule of mass m trapped in a spherical cage of radius *r*, the energy levels corresponding to the translational motion of the molecule are quantized [33,34] with energy $E_{l,n}=\beta_{l,n}^{2}{ћ}^{2}/2mr^{2}$ where *β_l,n_* is nth root of the *l_th_* order spherical Bessel function. As illustrated in Figure 8, the energy levels for a 15 Å cage for *m* = 3 are of the order of a few K and should be observable.

The spherical cage is a very poor approximation to the mesoporous cage of zeolite, which has several open channels but the model does provide an order of magnitude estimate for the translational energies. The values observed experimentally are indeed comparable to those expected in this model. In addition the energies appear to decrease as the filling is reduced and this is attributed to an effective increase in the bounding dimension for the lower *x* values with reduction of the blocking of the tunnels to the cages and, thus, leading to a decrease in the expected energies. These estimates are very approximate, and computer simulations for the zeolite geometries could provide a much better fit to the observed features.

**Figure 9:**
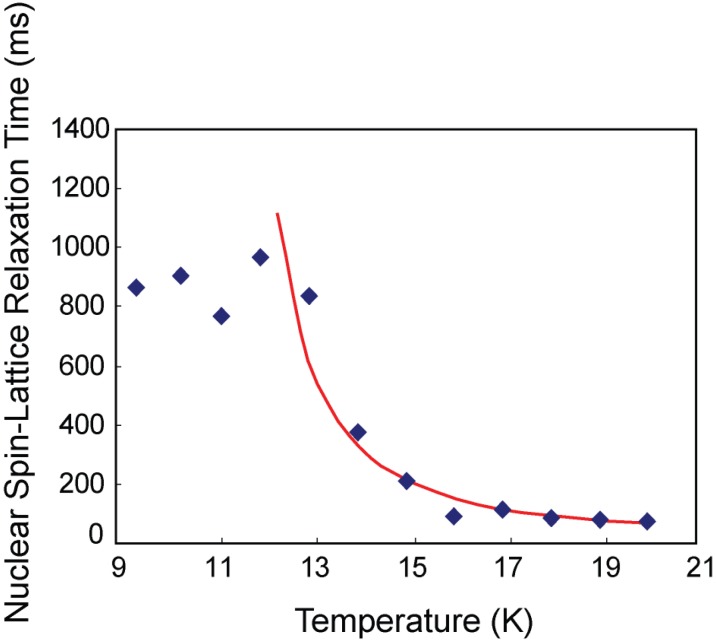
(Color on line) Variation of the nuclear spin-lattice relaxation time with temperature at high temperatures. The solid line (red) is a fit for a thermal activation of 73 ± 3 K and an intrinsic turneling rate of 1.5 × 10^11^ s^−1^.

It is noteworthy that, despite the high values for the absorption energy of the S1 and S2 sites, the molecules are still highly mobile. The relaxation times decrease exponentially below 12 K (Figure 9), according to a thermal activation process. The tunneling rate $\tau_{T}^{-1}=\tau_{0}^{-1}\exp(-E_{A}/k_{B}T)$ where the activation energy $E_{A}/k_{B}=73\pm 3$ K and *τ_0_* = 1.5 × 10^−11^ s^−1^. These values lead to a diffusion rate of (8.2 ± 2.8) × 10^−6^ cm^2^ s^−1^ at T = 19.5 K, which is to be compared with the value of 4.5 × 10^−6^ cm^2^ s^−1^ obtained at 17.4 K from the recent inelastic neutron scattering measurements of Coulomb *et al.* [35]. The small difference is accounted for by the small difference in temperatures of the two measurements and the experimental errors. The important feature of these results is that they clearly show that there exists a high mobility for low densities of hydrogen in zeolite down to very low temperatures.

## 4. Conclusions

Experimental investigations of the temperature variation of the proton spin-lattice relaxation time have shown the existence of distinctive peaks in the relaxation times for both CH_4_ and hydrogen deuteride molecules confined to the microporous cages of zeolite for concentrations equal to or less than one molecule per cage. This temperature dependence is interpreted in terms of discrete low energy excitations for the molecules. The origin of the excitations is different for CH_4_ and hydrogen deuteride. By using a well-known description of the coupling between different energy baths corresonding to these excitations, the peaks in the relaxation times are related to the peaks in the heat capacities of the relevant baths contributed by these excitations. This analysis is only valid when bottlenecks occur in the energy flow between the baths due to the weak couplings.

For the case of CH_4_ the levels are interpreted in terms of excited states for the *T*-rotational states of CH_4_. For hydrogen deuteride, the inferred energy levels are comparable to the values estimated for the quantized translational levels of molecules limited to motion inside spherical cages of 15 Å in diameter. Detailed theoretical calculations for the molecular motion in the connected cages of Zeolite like structures are needed to verify this interpretation. The results also show a strikingly high mobility for hydrogen molecule in these structures down to T ~ 10 K in agreement with recent neutron scattering studies.

## Figures and Tables

**Figure A1:**
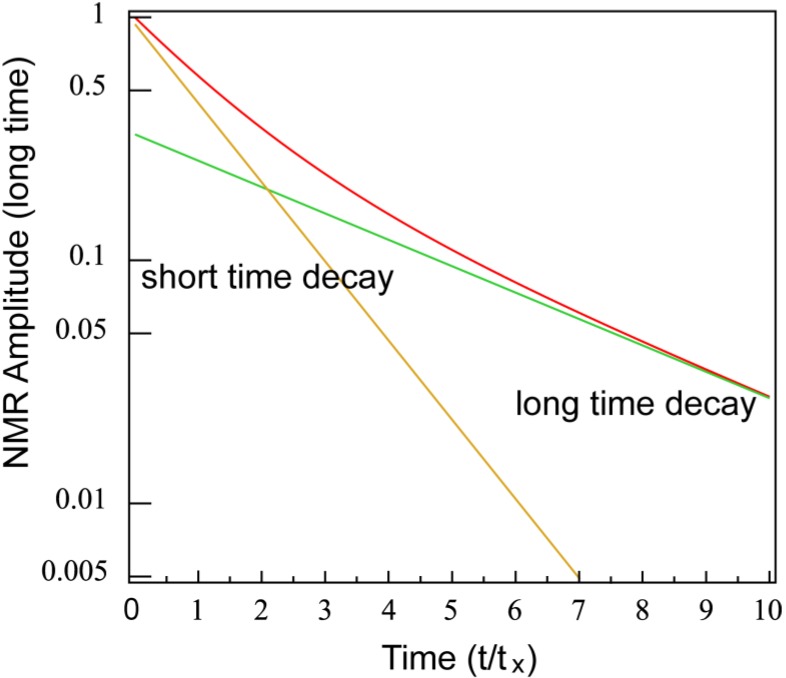
Schematic representation of the two decay rates for the three-bath system for μ=3 and $R_{AB}=R_{BL}=\tau_{X}^{-1}$ in Equation (A19). The short and long time decays are given by 1.33 $\tau_{X}$ and 4.0 $\tau_{X}$ , respectively.

**Figure A2:**
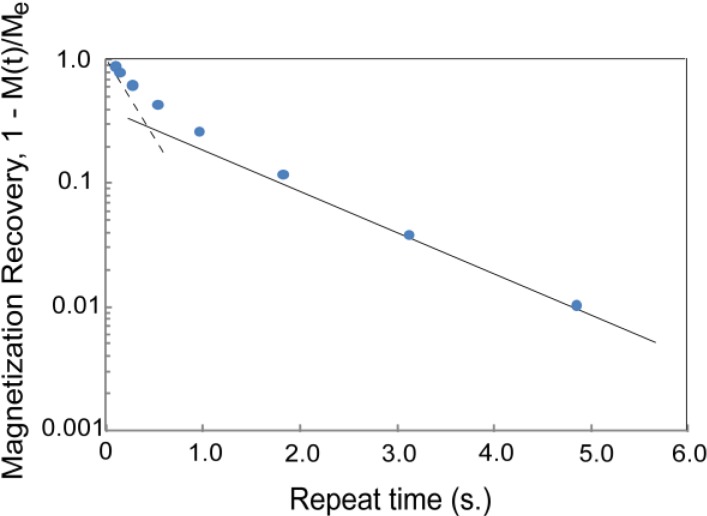
Observed relaxation for hydrogen deuteride molecules in zeolite-13X for 1 molecule per sodalite cage at 5.0 K. Two distinct relaxation times are observed, corresponding to the two-bath relaxation scenario.

**Figure B1:**
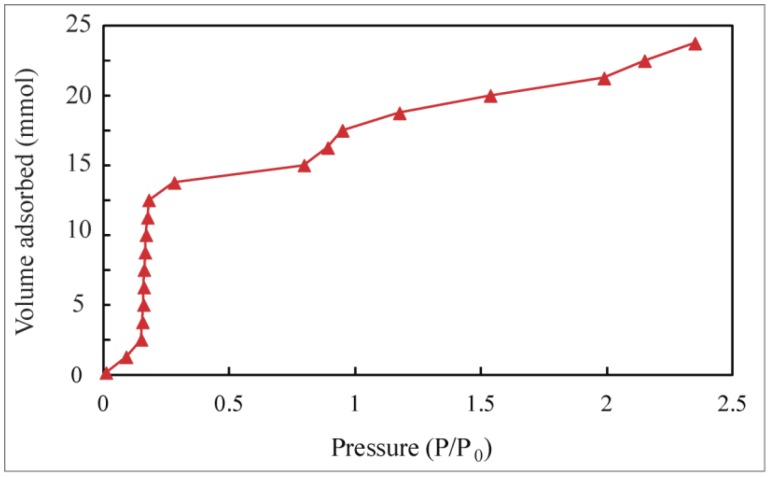
Adsorption isotherm for methane on zeolite-13X at 77 K. P_0_ = 17 mbar.

## Appendix A

### **Theoretical Treatment of Relaxation of Coupled Energy Baths**

For the three baths, A, B, and C of Figure 1, the total Hamiltonian for the complete system is written as

(A1)
$$H\;=\;H_{A}+H_{B}+H_{C}+H_{AB}+H_{BC}$$

where *H_A_*, *H_B_* and *H_C_* are the Hamiltonians for systems A, B and C respectively, and *H_AB_* and *H_BC_* represents the interaction between A and B, and B and C, respectively. It is assumed that the three baths are in thermal equilibrium and have only very weak couplings *H_AB_* and *H_BC_*, *i.e.*, [*H_A_*,*H_B_*] = 0 while $[H_{A},H_{AB}]\neq 0$ and $[H_{B},H_{BC}]\neq 0$ , and similarly for *H_B_* and *H_C_*. The evolution of A following a perturbation from equilibrium (e.g., by the application of an RF pulse) is determined by

(A2)
$$\frac{d}{dt}<H_{A}>\;=\;\frac{d}{dt}Tr(H_{A}\sigma )=Tr[\frac{\partial \sigma }{\partial t}H_{A}]$$

where σ is the spin density operator. The calculation can be developed following the description of Abragam [36] using the interaction representation (to remove the trivial time-dependence of the Larmor frequency) with $\frac{\partial \sigma^{*}}{\partial t}=[H_{AB},\sigma^{*}]$ .

(A3)
$$\frac{d}{dt}<H_{A}>\;=\;-{ћ}^{2}\int_{0}^{\text{∞}}d\tau Tr[H_{A}<[H_{AB}^{*}(\tau ),[H_{AB}^{*}(0),(\sigma^{*}-\sigma_{0})]]>$$

where *σ*_0_ is the equilibrium density matrix. The next step is to assume that each bath can be described by a spin temperature, or inverse spin temperature $\beta_{A}^{-1}=k_{B}T_{A}(t)$ : *i.e.*,

(A4)
$$\sigma^{*}\;=\;\exp[-\beta_{A}H_{A}]\exp[-\beta_{B}H_{B}].$$

As the B-system approaches the temperature of the A-system the time evolution is given by

(A5)
$$\sigma^{*}(t)\;\approx \;[1-\beta_{A}(t)-\beta_{B}(t)]\sigma_{0}(\beta_{A})$$

where *σ*_0_(*β_A_*) is the density operator for *H_A_* for spin temperature $\beta_{A}^{-1}$ . Using Equation (A5) in Equation (A3) we have

(A6)
$$\frac{d}{dt}<H_{A}>\;=\;[\beta_{A}(t)-\beta_{B}]{ћ}^{2}\int_{0}^{\text{∞}}d\tau <H_{A}(t)[H_{AB}^{*}(t),[H_{AB}^{*}(\tau ),H_{A}(t)]]>$$

where the average $<......>\;=\;Tr[\exp(-\beta H_{0}).....]$ . From Equation (A4) we can also write

$$\frac{d}{dt}<H_{A}>\;=\;-\beta_{A}(t)<H_{A}^{2}>\;=\;<{\left(H_{A}-<H_{A}>\right)}^{2}.$$

Noting that the heat capacity is given by $C_{A}=\frac{d}{dt}<H_{A}>=\frac{d\beta }{dT}\frac{d\langle H_{A}\rangle }{d\beta }=k_{A}\frac{d\beta }{dT}$ , we can define a heat capacity constant $k_{A}\;=\;\frac{dE_{A}}{d\beta }$ and similarly for *k_B_*.

The cross-relaxation rate $T_{AB}^{-1}$ between the A and the B reservoir is defined by $\frac{\partial \beta_{A}}{\partial t}\;=\;-T_{AB}^{-1}(\beta_{A}\left(t\right)-\beta_{B})$ and using Equation (A6) we find

(A7)
$$T_{AB}^{-1}\;=\;{ћ}^{2}k_{A}\int_{0}^{\text{∞}}d\tau <H_{A}(0)[H_{AB}^{*},(0),[H_{AB}^{*}(\tau ),H_{A}(0)]]>$$

From energy conservation $\frac{d}{dt}<H_{A}+H_{B}>\;=\;0$ and using

$$\frac{d}{dt}<H_{A}>\;=\;k_{A}\frac{\partial \beta_{A}}{\partial t}\text{ and }\frac{d}{dt}<H_{A}>\;=\;k_{B}\frac{\partial \beta_{B}}{\partial t}$$

we can employ $\frac{\partial }{\partial t}\beta_{B}\;=\;-T_{BA}^{-1}(\beta_{B}-\beta_{A})$ to show that

(A8)
$$T_{BA}^{-1}\;=\;T_{AB}^{-1}\frac{k_{A}}{k_{B}}\text{ or }R_{BA}\;=\;R_{AB}\frac{k_{A}}{k_{B}}$$

where $R_{AB}\;=\;T_{AB}^{-1}$ and similarly for *R_BA_*.

Equation (A8) is a very important result for the analysis of the experimental results discussed below because the total heat capacity $k_{A}+k_{B}$ and its components can be very different depending on the relevant degrees of freedom for the different energy baths and their temperature dependence. This dependence on the heat capacities will lead to significant changes in the observed relaxation rates and these changes can be used in turn to measure the excitation energies associated with the different modes that determine the heat capacities of the energy baths; hence the term, ***nuclear relaxation spectroscopy***. Examples of this dependence are the relaxation rates for different molecular species such as the ortho and para species in hydrogen, deuterium [13,14] and in methane [17].

For the three-bath system we have two coupled linear differential equations:

(A9a)
$$\frac{\partial \beta_{A}}{\partial t}=-R_{AB}(\beta_{A}-\beta_{B})$$

(A9b)
$$\frac{\partial \beta_{B}}{\partial t}\;=\;-R_{BA}(\beta_{B}-\beta_{A})-R_{BC}(\beta_{B}-\beta_{C})$$

(A9c)
$$\frac{\partial \beta_{C}}{\partial t}\;=\;-R_{CB}(\beta_{C}-\beta_{B})-R_{CL}(\beta_{C}-\beta_{L})$$

Using $R_{BA}\;=\;\frac{k_{A}}{k_{B}}R_{BA}$ we can write Equation (A9b) as

(A10)
$$\frac{\partial \beta_{B}}{\partial t}\;=\;-\frac{k_{A}}{k_{B}}R_{AB}(\beta_{B}-\beta_{A})-R_{BC}(\beta_{B}-\beta_{C})$$

and if C reaches equilibrium rapidly compared to the bottleneck between B and C, Equation (A9c) becomes $\frac{\partial \beta_{C}}{\partial t}=0$ .

We now consider the case for which baths A and B come rapidly to a common temperature, allowing one to express Equation (A9a) as $\beta_{B}-\beta_{A}=\frac{1}{R_{AB}}\frac{\partial \beta_{A}}{\partial t}$ . Inserting this relation in Equation (A10) we have

(A11)
$$\frac{\partial \beta_{B}}{\partial t}\;=\;-\frac{R_{BA}}{R_{AB}}\frac{\partial \beta_{B}}{\partial t}-R_{BC}(\beta_{B}-\beta_{C})$$

Now following the premise that the link BC is the weakest, that is that A and B reach equilibrium much faster than B and C, Equation (A11) can be rewritten as

(A12)
$$\frac{\partial \beta_{B}}{\partial t}[1+\frac{R_{BA}}{R_{AB}}]\;=\;-R_{BC}(\beta_{B}-\beta_{C})$$

The relaxation rate for B, the longest time constant in the system, and thus the effective observed relaxation rate is given by

(A13)
$$R_{eff}\;=\;R_{BC}/[1+k_{A}/k_{B}]$$

where we have used *k_B_R_BA_* = *k_A_R_AB_*. Equivalently, the effective nuclear spin relaxation time is

(A14)
$$\tau_{eff}\;=\;[(k_{A}+k_{B})/k_{B}]\tau_{X}$$

where $\tau_{X}\;=\;R_{BC}^{-1}$ is the cross-correlation time between baths B and C.

As noted by Guyer, Richardson and Zane [13], if the coupling of the baths A and B is slower than the coupling between B and C, then instead of Equation (A14) one observes $\tau_{eff}\;=\;R_{AB}^{-1}$ . The analysis also applies when bath C and the thermal reservoir are merged into one bath, except now Equation (A14) becomes

(A15)
$$\tau_{eff}\;=\;[(k_{A}+k_{B})/k_{B}]\tau_{BL}$$

where *τ_BL_* is the coupling of B to the lattice. The important point is that in both cases where there is a bottleneck between two baths, the effective relaxation time depends on the heat capacities (Equations (A14) and (A15), and thus measurements of the temperature dependence can be used to determine *k_A_* and *k_B_* and thus the excitation spectrum.

In most cases one observes in addition to the principal relaxation given by Equations (A14) and (A15) a smaller shorter (or sometimes slower) component to the relaxation. In order to analyze this feature we need to go beyond the treatment of Guyer, Richardson and Zane and consider more general time dependences. We write

(A16a)
$$\beta_{A}^{e}-\beta_{A}(t)\;=\;\xi_{A}e^{-R_{+}t}+\eta_{A}e^{-R_{-}t}$$

and

(A17b)
$$\beta_{B}^{e}-\beta_{B}(t)\;=\;\xi_{B}e^{-R_{+}t}+\eta_{B}e^{-R_{-}t}$$

where $\beta_{A}^{e}$ and $\beta_{B}^{e}$ are the equilibrium inverse temperatures. The initial conditions are

$$\beta_{A}^{0}\;=\;\beta_{A}^{e}-(\xi_{A}+\eta_{A})\text{ and }\beta_{A}^{0}\;=\;\beta_{A}^{e}-(\xi_{A}+\eta_{A}).$$

We are principally interested in the case where system B is weakly coupled to either another bath or to the lattice. Substituting Equation (A16) in the rate equations we find for the rate constants

(A18)
$$R_{\pm }\;=\;\frac{1}{2}\{(R_{AB}+R_{BA}+R_{AL}+R_{BL})\pm \sqrt{\left[{\left(R_{AB}+R_{BA}\right)}^{2}+{\left(R_{AL}-R_{BL}\right)}^{2}+2(R_{AB}-R_{BA})(R_{AL}-R_{BL})\right]}\}$$

and for the amplitudes we have

(A18a)
$$\xi_{A}\;=\;\frac{R_{AL}+R_{AB}-R_{-}}{R_{+}-R_{-}}(\beta_{A}^{e}-\beta_{A}^{0})-\frac{R_{AB}}{R_{+}-R_{-}}(\beta_{B}^{e}-\beta_{B}^{0})$$

(A18b)
$$\eta_{A}=\;\frac{R_{+}-R_{AL}-R_{AB}}{R_{+}-R_{-}}(\beta_{A}^{e}-\beta_{A}^{0})+\frac{R_{AB}}{R_{+}-R_{-}}(\beta_{B}^{e}-\beta_{B}^{0})$$

(A18c)
$$\xi_{B}=\;\frac{R_{AL}+R_{AB}-R_{-}}{R_{+}-R_{-}}(\beta_{A}^{e}-\beta_{A}^{0})-\frac{R_{AB}+R_{AL}-R_{+}}{R_{+}-R_{-}}(\beta_{B}^{e}-\beta_{B}^{0})$$

and

(A18d)
$$\eta_{B}=\;\frac{R_{+}-R_{AL}-R_{AB}}{R_{+}-R_{-}}(\beta_{A}^{e}-\beta_{A}^{0})+\frac{R_{AB}+R_{AL}-R_{-}}{R_{+}-R_{-}}(\beta_{B}^{e}-\beta_{B}^{0})$$

Note that $\xi_{A}+\eta_{A}=\beta_{A}^{e}-\beta_{A}^{0}$ and $\xi_{B}+\eta_{B}=\beta_{B}^{e}-\beta_{B}^{0}$ as required.

Defining $\mu =\;\frac{R_{BA}}{R_{AB}}=\;\frac{k_{A}}{k_{B}}$ , the ratio of the heat capacities, we examine two limiting cases that are particularly relevant to the experiments under discussion:

(i) Bath B isolated from the lattice but strongly coupled to bath (A), *i.e.*,
  $$R_{AB}>R_{AL}>>R_{BL},\text{ and}$$
(ii) Bath A more strongly coupled to the lattice than to bath B, *i.e.*,
  $$R_{AL}>R_{AB}>>R_{BL}.$$

In case (i) there is an initial fast relaxation given by the relaxation time

(A19a)
$$R_{+}=\;\frac{1+\mu }{\mu }R_{AB}[1+\frac{R_{BL}}{R_{AB}}\frac{1}{{\left(1+\mu \right)}^{2}}+......]$$

followed by a long time relaxation given by

(A19b)
$$R_{-}=\;\frac{R_{BL}}{1+\mu }[1-\frac{R_{BL}}{R_{AB}}\frac{1}{{\left(1+\mu \right)}^{2}}+.....]$$

where the relaxation times $\tau_{a}=R_{a}^{-1}$ *etc.* are in agreement with Table V of [12]. The corresponding amplitudes depend on the initial conditions and we will examine the practical case for which the A spins are saturated $\left(\beta_{A}^{0}=0\right)$ and the B spins are close to thermal equilibrium $\beta_{B}^{0}=\beta_{B}^{e}$ . The recovery of the two A spin amplitudes after saturation is given by

(a) $\xi_{A}≅\;\frac{\mu }{1+\mu }\beta_{A}^{e}\approx \;(1-\frac{1}{\mu })\beta_{A}^{e}$ , for the fast relaxing component, and
(b) $\eta_{A}\;≅\;\frac{1}{1+\mu }\beta_{A}^{e}\approx \;\frac{1}{\mu }\beta_{A}^{e}$ , for the slow relaxing component.

In the cases of interest in this study, μ>1 and $\xi_{A}>\eta_{A}$ . Figure A1 shows a schematic illustration of this recovery for μ>1 .

The significance is that the rates and the amplitudes both depend on the ratio of the heat capacities (or the magnetizations if the nuclear species are different). This is the principal point for the studies reported here where the cross-relaxation and lattice relaxation times are constant or well-known slowly varying functions. The observed relaxation will depend strongly on the specific heats capacities of the thermal baths and measurements of the relaxation times can determine the relevant specific heats. (For reference the reader should note that while Guyer, Richardson and Zane [13] derive similar rate equations (see their Appendix A), they do not calculate the amplitudes and only give the rates to first order).

In case (ii) for which $R_{BL}>R_{BA}>>R_{AL}$ , the relaxation times are given by a short term relaxation:

(A20a)
$$\tau_{-}=\tau_{BL}[1-\frac{\tau_{BL}}{\tau_{BA}}+....]$$

followed by a long term relaxation;

(A21a)
$$\tau_{+}=\;\frac{\tau_{BA}}{\mu }[1+\frac{\tau_{BL}}{\tau_{BA}}+.....]$$

The amplitudes of the fast and long time relaxations for recovery following saturation of the B spins for the case $R_{AL}>R_{AB}R_{BL}$ (or $\tau_{AL}<\tau_{AB}<\tau_{BL}$ ) are given respectively by:

(a) $\xi_{A}=\;{\left(\frac{\tau_{BL}}{\tau_{BA}}\right)}^{2}\beta_{A}^{e}<<\beta_{A}^{e}$ , and
(b) $\eta_{A}≅\;[1-{\left(\frac{\tau_{BL}}{\tau_{BA}}\right)}^{2}+...]\beta_{A}^{e}\approx \beta_{A}^{e}$ .

Understanding the origin of the two components for the relaxation is important for the analysis of the data. As an example we show in Figure A2 the relaxation observed for HD molecules at 5 K where two components are clearly visible. The ratio of the amplitudes can also be used to determine µ, the ratio of the heat capacities of the baths.

## Appendix B

### **Adsorption Isotherms**

The adsorption isotherms for methane on zeolite-13X were determined by measuring the equilibrium gas pressure after adsorption on the zeolite in the NMR cell as a function of known amounts of added gas at a temperature of 77 K. A typical isotherm is shown in Figure B1. The knee at 1.25 × 10^−2^ mole adsorbed gas corresponds to a saturated monolayer or an adsorption area of 603 m^2^/g for the zeolite sample. This adsorption value is attributed to saturation of the S1 sites of the α-supercage of zeolite. The second knee at 1.55 × 10^−2^ mole corresponds to 748 m^2^/g which is to be compared with the values in the literature of 680–800 m^2^/g for the total available surface area reported in the literature for zeolite-13X. [37,38,39]

## References

[1] Eddaoudi M., Kim J., Rosi N., Vodak D., Wachter J., O’Keefe M., Yaghi O.M. (2002). Systematic design of pore size and functionality in isoreticular MOFs and their application in methane storage. Science.

[2] Alavi S. (2010). Selective guest docking in metal-organic framework materials. ChemPhysChem.

[3] Yildirim T., Hartman M.R. (2005). Direct observation of hydrogen adsorption sites and nanocage formation in metal-organic frameworks. Phys. Rev. Lett..

[4] Jansen J.C., Stoecker M., Kange H.G., Weitkamp J. (1994). Advanced Zeolite Science and Applications, Volume 85 (Studies in Surface Science and Catalysis.

[5] Ma S., Sun D., Simmons J.M., Collier C.D., Yuan D., Zhou H.-C. (2008). Metal-Organic framework from an anthracene derivative containing nanoscopic cages exhibiting high methane uptake. J. Am. Chem. Soc..

[6] (2003). Basic research needs for the Hydrogen Economy: Workshop on Hydrogen Production, Storage and Use. http://www.sc.doe.gov/bes/hydrogen.pdf.

[7] Pang J., Hampsey J.E., Wu Z., Hu Q., Lu Y. (2004). Hydrogen adsorption in mesoporous carbons. Appl. Phys. Lett..

[8] Giraudet S., Zhu Z. (2011). hydrogen adsorption in nitrogen enriched ordered mesoporous carbons doped with nickel nanoparticles. Carbon.

[9] Yuan F., Li G. (2005). Low temperature catalytic conversion of methane to methanol by barium sulfate nanotubes supporting sulfates: Pt(SO_4_)_2_, HgSO_4_, Ce(SO_4_)_2_ and Pb(SO_4_)_2_. Chem. Commun..

[10] Matsushita T., Toda R., Hieda M., Wada N. (2010). Quantum states of helium atoms confined in nanocage in Na-Y zeolite. J. Low Temp. Phys..

[11] Wada N., Cole M. (2008). Low-Dimensional helium quantum fluids realized under nano-extreme conditions. J. Phys. Soc. Jpn..

[12] Jee S.E., McGaughey A.J.H., Scoll D.S. (2008). Molecular simulations of hydrogen and methane permeation through pore mouth modified zeolite membranes. Mol. Simul..

[13] Guyer R.A., Richardson R.C., Zane L.I. (1971). Excitations in quantum crystals (A Survey of NMR Experiments in Solid Helium). Rev. Mod. Phys..

[14] Weinhaus F., Meyer H., Myers S.M., Harris A.B. (1971). Nuclear longitudinal relaxation in HCP D_2_. Phys. Rev..

[15] Zhou D., Rall M., Brison J.P., Sullivan N.S. (1990). NMR studies of vacancy motion in solid hydrogen. Phys. Rev..

[16] Rall M., Zhou D., Kisvarsanyi E.G., Sullivan N.S. (1992). Nuclear spin-spin relaxation of isotopic impurities in solid hydrogen. Phys. Rev..

[17] Nijman L. (1977). Investigation of Solid Methane by NMR at High Pressure and Low Temperature. Ph.D. Thesis.

[18] Zeolite-13X, Part number MS-1343.

[19] Green S. (2009). Nitrogen gas storage in a zeolite. Research Experience for Undergraduates Report, National High. Magnetic Field Laboratory. http://www.magnet.fsu.edu/education/reu/program/2009/documents/green.pdf.

[20] Teflon E.I.

[21] Ji Y., Hamida J.A., Sullivan N.S. (2010). NMR Studies of Quantum Rotors Confined in Zeolite. J. Low Temp. Phys..

[22] Kumar A.V.A., Yashonath S., Sluiter M., Kawazoe Y. (2001). Rotational motion of methane within the confines of zeolite NaCaA: Molecular dynamics and *ab initio* calculations. Phys. Rev..

[23] Bloembergen N., Purcell E.M., Pound R.V. (1948). Relaxation effects in nuclear magnetic resonance absorption. Phys. Rev..

[24] Sullivan N.S., Deschamps P., Neel P., Vaisiere J.M. (1983). Efficient fast-recovery scheme for NMR pulse spectrometers. Revue Phys. Appl..

[25] Genio E.B., Ihas G.G., Sullivan N.S. (1998). Ultra-Low temperature thermometry using zeeman perturbed NQR. J. Low Temp. Phys..

[26] Stroud H.J.F., Richards E., Limcharden P., Parsonage N.F. (1976). Thermodynamic study of the linde sieve 5A + methane system. JCS Faraday Trans..

[27] Yashonanth S., Thomas J.M., Nowak K., Cheetham A.K. (1988). The siting, energetics and mobility of saturated hydrocarbons inside zeolitic cages: Methane in zeolite Y. Nature.

[28] Sullivan N.S. (1988). Orientational order–disorder transitions in solid hydrogen. Can. J. Chem..

[29] DeWall J., Dimeno R.M., Sokol P.E. (2002). Slow diffusion of molecular hydrogen in zeolite. J. Low Temp. Phys..

[30] Eckert J., Nichol J.M., Howard J., Trouw F.R. (1996). Adsorption of hydrogen in Ca^2+^ exchanged zeolite A probed by inelastic neutron scattering, spectroscopy. J. Phys. Chem..

[31] Pulido A., Nachtigall P., Delgado M.R., Arean C.O. (2009). Computational and variable-temperature infrared spectroscopic studies on carbon monoxide adsorption on Zeolite Ca-A. ChemPhysChem.

[32] Arean C.O., Delgado M.R., Bauca C.L., Vrbyka L., Nachtigall P. (2007). Carbon monoxide adsorption on low-silica zeolites—From single to dual and to multiple cation sites. Phys. Chem. Chem. Phys..

[33] Gasiorowicz S. (1974). The Radial Equation, Quantum Physics.

[34] Liboff R.L. (1998). The Free Particle Wavefunction, Introductory Quantum Mechanics.

[35] Coulomb J.P., Flouquet N., Dufau N., Llewellyn P., Andre G. (2007). Structural and dynamic properties of confined hydrogen isotopes (H2, HD, D2) in model porous materials: Silicalite-I, AlPO4-N family (N = 5, 8.11, 54) and MCM-41 (Φ = 25 Å). Microporous Mesoporous Mater..

[36] Abragam A. (1961). The Principles of Nuclear Magnetism.

[37] Yates D.J.C. (1968). Studies of the surface area of Zeolites. Can. J. Chem..

[38] Vyas R.K., Kumar S.H., Kumar S.U. (2004). Determination of volume and surface area of zeolite molecular sieves by D_R and D_A equations—A comparative study. Indian J. Chem. Technol..

[39] Akhtar F., Bergstrom L. (2011). Colloidal processing and thermal treatment of binderless hierarchically porous Zeolite-13X Monoliths for CO_2_ capture. J. Am. Ceram. Soc..

